# Preparation of Unburned Lightweight Aggregates via Synergistic Utilization of Red Mud and Multi-Source Solid Wastes and Its Performance Investigation

**DOI:** 10.3390/ma19122490

**Published:** 2026-06-10

**Authors:** Jixiang Cai, Lianghuan Wei, Xianghao Zha, Rubin Han, Hui Luo

**Affiliations:** 1 Xinjiang Biomass Solid Waste Resources Technology and Engineering Center, College of Chemistry and Environmental Science, Kashi University, Kashi 844000, China; caijixiangedukashi@163.com; 2 School of Civil and Ocean Engineering, Jiangsu Ocean University, Lianyungang 222005, China; 19816221775@163.com (R.H.); water@njfu.edu.cn (H.L.)

**Keywords:** red mud, fly ash, physical properties, microscopic analysis, environmental safety

## Abstract

**Highlights:**

- RMLWA was fabricated from red mud, fly ash, and granulated blast-furnace slag using alkali-activated technology.
- The physical properties and microstructure of RMLWA were analyzed.
- The heavy metal leaching characteristics of RMLWA were analyzed.

**Abstract:**

This study aims to explore the preparation process and properties of unburned lightweight aggregate using red mud synergistically with fly ash, granulated blast-furnace slag, and other multi-source solid wastes. Curing regimes and alkali-activated systems were controlled. Their effects on physical properties and environmental safety of lightweight aggregate were systematically evaluated. Results show that curing temperature and alkali activator exert significant synergistic effects on physical properties of lightweight aggregates. Steam curing performs better than standard curing. Performance improves with increasing steam temperature. Sodium silicate solution with a modulus of 1.0 is determined as the optimal activator. Under 90 °C steam curing, Sample D2 achieves the best overall performance. Its cylinder compressive strength reaches 6.92 MPa. 1 h water absorption is 14.8%. Softening coefficient is 0.93. Porosity is as low as 31.07%. Microscopic analysis reveals that higher curing temperature significantly accelerates the hydration reaction of the RMLWA system. It promotes the formation of abundant cementitious products such as C-S-H gel. These products fully fill internal pores and microcracks of the aggregate. A dense three-dimensional network skeleton structure is finally formed. For environmental safety, heavy metal leaching concentrations of steam-cured samples are generally lower than those of standard-cured samples. This study realizes high-value resource utilization of industrial solid wastes. It also provides a new technical route for the development of green building lightweight aggregate.

## 1. Introduction

With the continuous development of industrialization and urbanization, the generation of large amounts of industrial solid waste has become a key issue restricting the sustainable development of the ecological environment [1,2,3]. Among them, typical solid wastes such as red mud (RM), fly ash (FA), and granulated blast-furnace slag (GGBS) have attracted extensive attention due to their large output, difficult storage, and potential environmental risks [4,5]. Taking red mud as an example, its strong alkalinity and high heavy metal content lead to long-term stockpiling. This not only occupies a large amount of land resources but also may cause soil and groundwater pollution [6,7]. Therefore, exploring high-value synergistic utilization pathways for multi-source solid wastes is of great significance for resource recycling and environmental protection.

Artificial lightweight aggregate is an important building material. It has broad application prospects in lightweight concrete, prefabricated buildings, and sponge city construction due to its low density, adjustable strength, and good thermal insulation performance [8,9,10]. In recent years, preparing lightweight aggregate from industrial solid waste has gradually become a research hotspot. Traditional sintered lightweight aggregate can achieve high strength. However, its production usually relies on high-temperature calcination with high energy consumption and large carbon emissions [11,12]. This is inconsistent with the current “dual carbon” goals. In contrast, unburned lightweight aggregate is hardened through hydration or alkali-activated reactions. It has the advantages of low energy consumption, simple process, and environmental friendliness. It is regarded as a green preparation technology with great development potential.

Therefore, many researchers have conducted studies on unburned preparation processes. For example, Tang et al. [13] prepared unburned lightweight aggregate from solid waste with a compressive strength of 8.9 MPa, bulk density of 556 kg/m^3^, and 1 h water absorption of 5.9%. These properties meet the requirements of Lightweight Aggregates and Its Test Methods—Part 1: Lightweight Aggregates (GB/T 17431.1-2010) [14]. Ma et al. [15] used sewage sludge and fly ash as raw materials. The cylinder compressive strength of the prepared lightweight aggregate reached 7.43 MPa. Its apparent density, bulk density, and water absorption were 2603 kg/m^3^, 852 kg/m^3^, and 8.37%, respectively. In addition, Song et al. [16] prepared sintered lightweight aggregate from construction waste and fly ash. The optimal process parameters were determined as follows: construction waste/fly ash mass ratio of 5:5, calcination temperature of 1120 °C, and calcination time of 20 min. The ceramsite obtained under these conditions showed good performance. Its particle strength was 9.75 MPa, porosity was 61.09%, and 1 h water absorption was 14.95%. Although many studies have investigated the preparation and properties of solid waste-based lightweight aggregate, systematic research on the synergistic effects of curing regimes (especially steam curing temperature) and alkali-activated systems on aggregate performance is still insufficient. In addition, systematic evaluation of the environmental safety of red-mud-containing systems, such as heavy metal leaching behavior, needs to be further strengthened.

Previous studies have shown that fly ash and slag are typical silico-aluminous and calcium-silicate materials. They can form cementitious products such as C-S-H gel under alkali activation, thus providing good mechanical properties for materials [17]. However, single solid waste systems often suffer from insufficient reaction activity or poor structural stability. Red mud is rich in Fe_2_O_3_, Al_2_O_3_, and a certain quantity of alkaline components. Its introduction can not only supply aluminum sources but also participate in reactions in alkaline environments to improve the reaction activity of the system [6,18]. Therefore, constructing a multi-component synergistic system with red mud, fly ash, and slag is expected to achieve complementary advantages and improve the overall performance of lightweight aggregate.

Based on the above analysis, this study uses red mud, fly ash, and granulated blast-furnace slag as main raw materials. Unburned artificial lightweight aggregate (RMLWA) is prepared via pelletizing and curing processes. The effects of different curing regimes and alkali-activated systems on its physical properties are mainly discussed. Meanwhile, microstructural characteristics are systematically analyzed using scanning electron microscopy with energy-dispersive X-ray spectroscopy (SEM-EDS), X-ray diffraction (XRD), and Fourier-transform infrared spectroscopy (FTIR). The environmental safety of the material is evaluated via heavy metal leaching tests. This study can provide theoretical basis and technical support for the resource utilization of industrial solid waste and the development of green building materials.

## 2. Materials and Methods

### 2.1. Raw Materials

**Figure 1:**
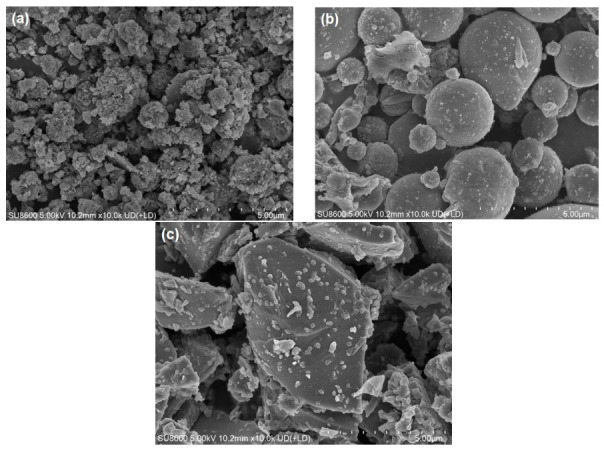
SEM images of RM (**a**), FA (**b**), and GGBS (**c**).

The raw materials used in this study mainly include red mud (RM), fly ash (FA), and granulated blast-furnace slag (GGBS). First, the chemical compositions of each raw material were determined by X-ray fluorescence spectroscopy (XRF), and the results are listed in Table 1. Meanwhile, their mineral compositions, micro-morphologies, and particle size distributions were analyzed using scanning electron microscopy (SEM) and laser particle size analyzer, with the relevant results shown in Figure 1.

The RM used in this experiment was obtained from a production enterprise in Liaocheng City, Shandong Province. Its main chemical components include Fe_2_O_3_, Al_2_O_3_, and SiO_2_. Its mineral phases are mainly hematite and quartz. Benefiting from the presence of hematite, the red mud presents an obvious red appearance. In addition, the total content of active components such as aluminum, silicon, and calcium in it exceeds 50%. This indicates that it has high potential cementitious activity. It can be observed from Figure 1a that the microstructure of red mud is relatively loose. It is mainly formed by the agglomeration of irregular particles. The internal pores of the agglomerates are well developed. This is the main reason for its large specific surface area, high water absorption, and excellent water retention performance.

FA was sourced from Xindian Fly Ash Co., Ltd. (Lianyungang, China). Its mineral composition is mainly quartz. Figure 1b shows that fly ash particles are spherical. Chemical analysis results indicate that the SiO_2_ content in fly ash is 48.12%. The total proportion of active components such as aluminum, silicon, and calcium exceeds 50%. This shows good cementitious potential. At the same time, its Al_2_O_3_ content is as high as 35.15%. It is rich in aluminum element and can be used as an aluminum source regulating material in the preparation process of lightweight aggregate.

GGBS was provided by Hanjiang Mining Technology Co., Ltd. (Lianyungang, China). Its main chemical components are CaO and SiO_2_. Its mineral composition is also mainly quartz. It can be observed from Figure 1c that its microstructure presents an irregular block structure.

The experimental water was laboratory tap water. The sodium silicate (Na_2_SiO_3_·9H_2_O) used was an analytical reagent (AR), purchased from Sinopharm Group Shanghai Co., Ltd. (Shanghai, China).

**Figure 2:**
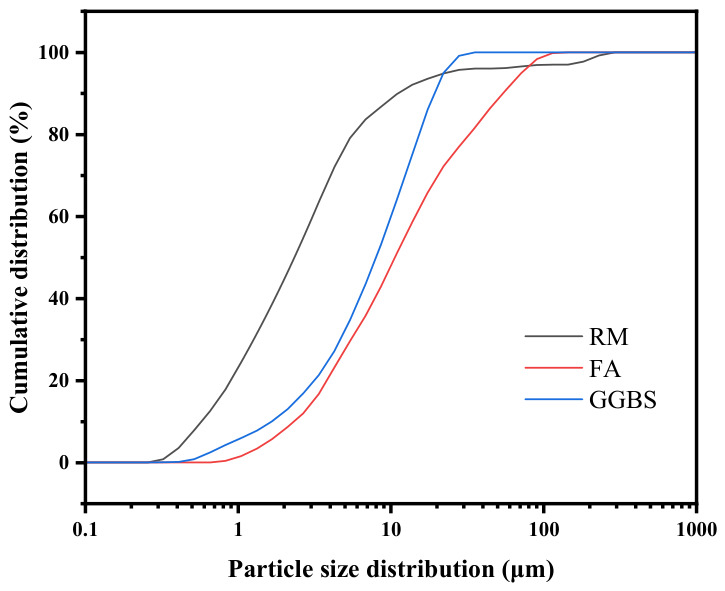
Particle size distribution plots of RM, FA, and GGBS.

In addition, Figure 2 shows the particle size distribution of each raw material. From the perspective of particle size distribution, RM (d_50_ = 2.339 μm), FA (d_50_ = 10.658 μm), and GGBS (d_50_ = 8.008 μm) have complementary particle size gradients. It is noteworthy that the three curves overlap in the coarse particle range of 20–100 μm, corresponding to a cumulative distribution above 90%. This phenomenon is attributed to the similar formation mechanisms of the three raw materials, the concentrated upper limit of coarse particles within this range, and the limited effect of uniform grinding pretreatment on coarse particle size. This gradient characteristic is conducive to the uniform mixing and dense forming of each raw material.

### 2.2. Preparation Method and Maintenance Conditions for RMLWA

Based on previous research and experimental results, the optimal mass ratio of RM, FA, and GGBS in the RMLWA system was determined to be 6:3:1. At the start of the test, RM, FA, and GGBS were weighed according to the designed ratio, placed in a blender, and mixed thoroughly for no less than 30 min to ensure uniform distribution of each component. Subsequently, the uniform mixture was fed into a disc granulator for pelletizing, with the equipment rotation speed controlled at 30 r/min. During the granulation process, tap water (TW), sodium silicate solution with a modulus of 1.0 (SS-1.0), and sodium silicate solution with a modulus of 2.0 (SS-2.0) were evenly sprayed on the surface of the material, so that their addition amount reached 30% of the dry mass of the raw materials. The entire granulation time was controlled within 20 min, and spherical particles with a particle size of 8–10 mm were finally prepared. This granulation process complied with the relevant requirements of Chinese Standard GB/T 17431.1-2010.

The formed RMLWA was first cured at room temperature (20 °C ± 2 °C) for 24 h. Then, it was treated according to different curing systems (see Table 2). Some samples were placed in a standard constant temperature and humidity curing box (SHBY-40B, produced by Shanghai Kanglu Instrument and Equipment Co., Ltd., Shanghai, China) for standard curing for 24 h, numbered A1–A3; the remaining samples were transferred to a steam curing box (ZKY-400B), and steam cured at 50 °C, 70 °C, and 90 °C for 24 h, respectively, under the condition of 99% relative humidity, corresponding to numbers B1–D3. After curing, all samples were placed in an electric blast drying oven (DHG9101) for drying for 12 h, and the final RMLWA products were obtained. The entire preparation process complied with the relevant specifications of Lightweight Aggregates and Their Test Methods (GB/T 17431.1-2010).

### 2.3. Test Methods

#### 2.3.1. Physical Property Testing

In the application of building materials, lightweight aggregates need to have certain mechanical properties and appropriate water absorption characteristics. Therefore, in this study, cylinder compressive strength, 1 h water absorption rate, and bulk density were selected as the main evaluation indicators, and the relevant test parameters and calculation formulas are listed in Table 3. To ensure the reliability and accuracy of the experimental results, all samples were measured in parallel three times, and the average value was taken.

#### 2.3.2. Microscopic Testing

In this study, the microstructure of industrial solid waste raw materials and lightweight aggregates was detected by scanning electron microscope (SEM, SM-7200; JEOL Ltd., Tokyo, Japan), and energy dispersive spectroscopy (EDS) was used to analyze the chemical composition of artificial lightweight aggregate products. X-ray diffraction (XRD; JEOL Ltd., Tokyo, Japan) was employed to identify the phase composition of artificial lightweight aggregates at different temperatures. Fourier transform infrared spectroscopy (FTIR; Thermo Fisher Scientific Inc., Waltham, MA, USA) test was used to analyze various functional groups in the range of 4000–400 cm^−1^.

#### 2.3.3. Heavy Metal Leaching Characteristic Testing

In this study, the heavy metal leaching characteristics of aggregates were determined by inductively coupled plasma optical emission spectrometry (ICP-OES). The testing was performed in accordance with the Chinese standard “Leaching Toxicity Test Method for Solid Wastes—Horizontal Oscillation Method” (HJ557-2010) [19].

## 3. Results and Discussion

### 3.1. Physical Properties of RMLWA

#### 3.1.1. Cylinder Compressive Strength

**Figure 3:**
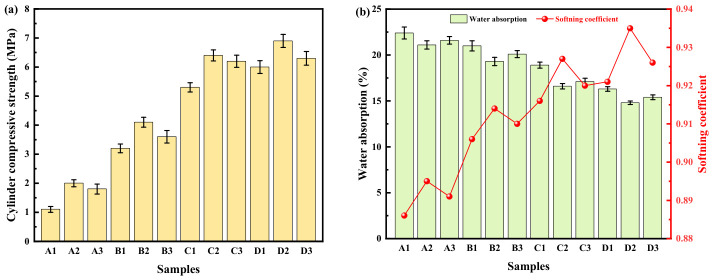
Cylinder compressive strength of RMLWA (**a**) Water absorption rate and softening coefficient (**b**).

Figure 3a shows the variation trend of cylinder compressive strength of RMLWA under different conditions. Overall, both curing temperature and liquid activator type had significant effects on the cylinder compressive strength of RMLWA, and the cylinder compressive strength of steam-cured samples was generally higher than that of standard-cured samples.

Under standard curing conditions, the cylinder compressive strength of sample A1 with water as additive was the lowest, only 1.12 MPa. After using sodium silicate solution as additive, the cylinder compressive strengths of samples A2 and A3 increased to 2.06 MPa and 1.85 MPa, respectively, indicating that sodium silicate had an obvious activating effect on the cementitious activity in RMLWA. This is because SiO_3_^2−^ dissociated from sodium silicate in aqueous solution can undergo hydration reactions with Ca^2+^ and Al^3+^ in raw materials to generate cementitious products such as C-S-H, which fill the internal pores of aggregates and improve structural compactness [20].

Under steam curing conditions, the cylinder compressive strength of samples increased significantly with the increase in temperature, among which sample D2 reached the peak with a cylinder compressive strength of 6.92 MPa, which was greatly higher than that of sample A2 under standard curing with the same additive. The reason is that properly increasing the steam-curing temperature can accelerate the hydration reaction rate of active SiO_2_, Al_2_O_3_, and CaO in raw materials, shorten the generation time of cementitious products, and make the internal structure of aggregates tend to be dense faster [21,22].

#### 3.1.2. 1 h Water Absorption Rate and Softening Coefficient

Figure 3b shows the changes in 1 h water absorption rate and softening coefficient of RMLWA under different conditions. It can be seen from the figure that under the same curing condition, the water absorption rate of samples prepared with TW was the highest, while that of samples prepared with SS was significantly lower. The 1 h water absorption rate of sample A1 under standard curing reached 22.4%, that of sample A2 prepared with SS-1.0 decreased to 21.1%, and that of sample A3 prepared with SS-2.0 was 21.6%. During the steam curing stage, the water absorption rate continued to decrease with the increase in temperature. The water absorption rate of sample D2 cured by steam at 90 °C decreased to 14.8%, which was the lowest among all samples. Compared with sample B2 with the same additive at 50 °C, it decreased by 23.31%; compared with sample C2 with the same activator at 70 °C, it decreased by 10.84%; compared with sample D3 at the same temperature, it decreased by 4.05%. This is because the water absorption rate of aggregates is directly related to the proportion of internal connected pores. When steam curing at 90 °C and sodium silicate solution with a modulus of 1.0 act synergistically, the hydration reaction of the system is the most sufficient, and the generated cementitious products are the largest in quantity and the most uniform in distribution, fully filling the connected pores inside the aggregates and effectively blocking the water infiltration channels [15,23]. In terms of softening coefficient, the softening coefficient of sample D2 reached 0.93, which was the highest among all samples, with the best water stability. This is because the sodium silicate solution better stimulated the activity of RMLWA, the amount of hydration products increased, and water was difficult to penetrate into the interior of aggregates to erode the cementitious skeleton, so the softening coefficient of aggregates was significantly improved [24]. Notably, the softening coefficient is governed jointly by hydration product stability, compactness, and pore connectivity [25,26]. Higher curing temperature greatly accelerates alkali-activated reactions, facilitating the formation of abundant C-S-H gel and dense three-dimensional networks [27,28]. The reduced connected pores improve water penetration resistance, minimize strength loss under saturation, and elevate the softening coefficient. For samples cured at the same temperature, sodium silicate at modulus 1.0 provides suitable alkalinity to fully activate fly ash and slag, forming C-S-H gel with low Ca/Si ratio and superior water stability [28,29]. In contrast, modulus 2.0 leads to excessive free silica and insufficient alkalinity, which hinders raw material depolymerization. The resultant hydration products are low in quantity and polymerization degree, and the increased micropores and microcracks allow easy water ingress to damage the cementitious skeleton, further reducing strength and softening coefficient under saturation. Standard curing leads to slow and incomplete hydration [30,31]. The loose structure with abundant connected pores is susceptible to water erosion, resulting in large strength loss and an overall low softening coefficient.

#### 3.1.3. Bulk Density, Apparent Density, and Void Ratio

**Figure 4:**
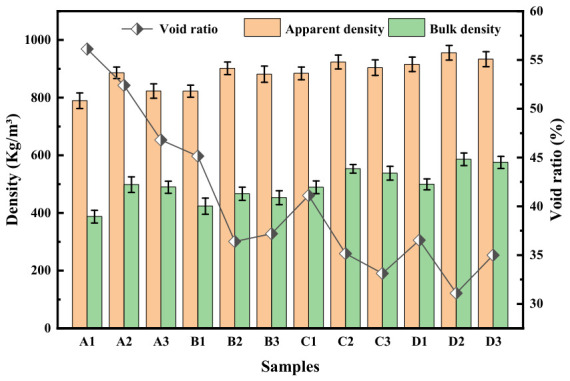
Apparent density, bulk density, and void ratio of RMLWA.

Figure 4 shows the variation rules of bulk density, apparent density, and void ratio of RMLWA under different conditions. It can be seen from the figure that the apparent density and bulk density of sample A1 under standard curing were the lowest, which were 789.41 kg/m^3^ and 387.22 kg/m^3^, respectively. The apparent densities of samples A2 and A3 prepared with sodium silicate solution increased to 886.34 kg/m^3^ and 823.17 kg/m^3^, and the bulk density of sample A2 increased to 498.15 kg/m^3^. In addition, after entering the steam curing stage, the apparent density and bulk density continued to increase, and the bulk density of sample D2 reached the highest among all samples, which was 586.21 kg/m^3^. Furthermore, the void ratio of sample A1 under standard curing was the highest, 56.13%, while the void ratio of sample A2 prepared with SS-1.0 decreased significantly. During the steam curing stage, the void ratio further decreased, and the void ratio of sample D2 was the lowest among all samples, 31.07%. This is because the hydration reaction was the most sufficient at this time, and a large number of generated cementitious products fully filled the internal pores and cracks of the aggregates, enhancing the structural compactness [32].

### 3.2. Microscopic Analysis of RMLWA

#### 3.2.1. SEM-EDS

**Figure 5:**
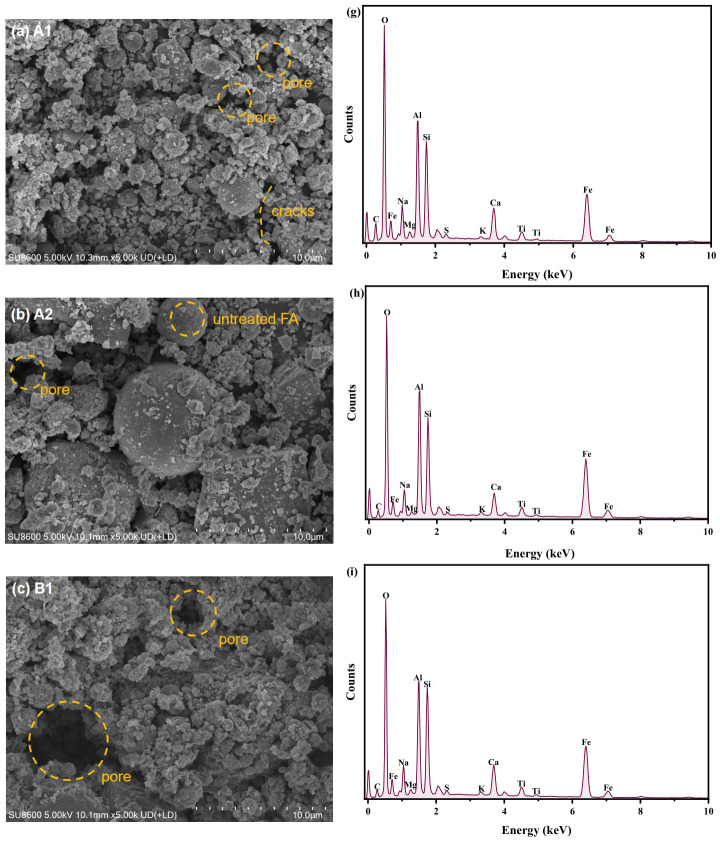

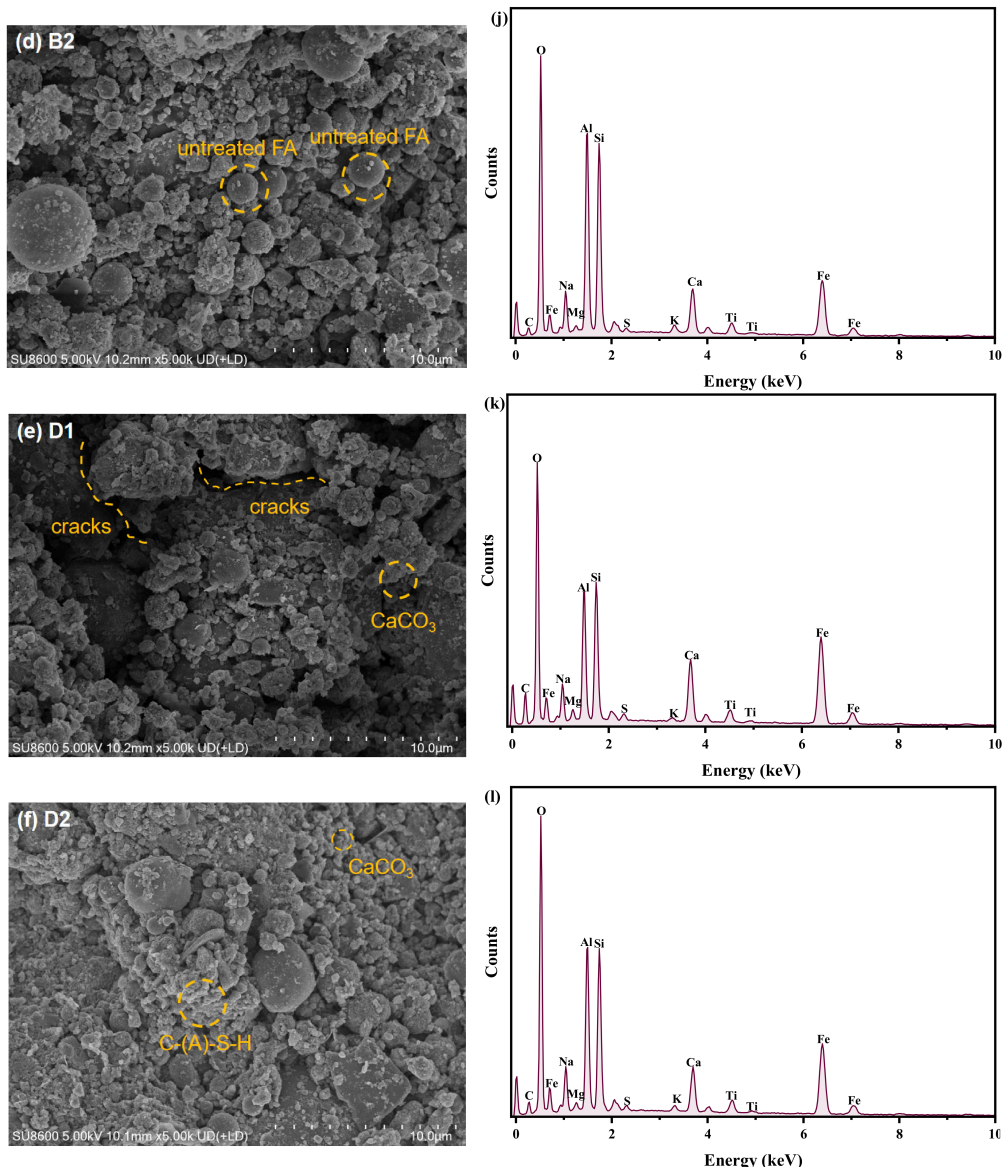
The SEM micrographs (**a**–**f**) and EDS (**g**–**l**) results of RMLWA.

SEM-EDS analysis was performed on six groups of samples (A1, A2, B1, B2, D1, D2) to explore the effects of curing conditions and activator types on the microstructure of RMLWA. For specific details, refer to Figure 5. The results showed that sample A1 under standard curing had a large number of connected pores and cracks inside, with a loose and porous structure. For sample A2 added with SS-1.0, a small quantity of flocculent hydration products adhered to the particle surface, the pores were partially filled, and the structural compactness was slightly improved. However, the hydration reaction was insufficient at room temperature, and there were still many micropores. For sample B1 cured by steam at 50 °C, low-temperature steam slightly promoted hydration, a small amount of hydration products appeared between particles, and the pores were slightly reduced, but the overall structure was still relatively loose. Under the synergistic effect of SS-1.0 and 50 °C steam, the hydration reaction of sample B2 accelerated, the particle surface was wrapped by a large number of flocculent hydration products, and the connected pores were greatly reduced. For sample D1 cured by steam at 90 °C, high-temperature steam further promoted the hydration reaction, a thin gel layer was formed between particles, and the pores were further reduced. However, the quantity of cementitious products generated was limited without the action of activator, and there were still some connected pores. In contrast, under the synergistic effect of 90 °C steam and SS-1.0, the hydration reaction of sample D2 was the most sufficient. The raw material particles were completely wrapped by a large number of dense hydration products, and the pores and microcracks between particles were fully filled by hydration products, forming a continuous and dense three-dimensional network skeleton structure [33].

#### 3.2.2. XRD

**Figure 6:**
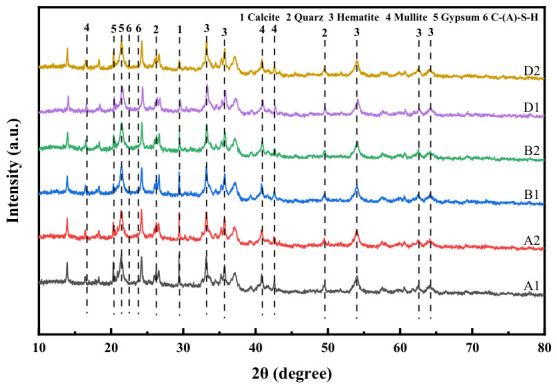
XRD pattern of RMLWA.

Figure 6 shows the XRD results of six groups of samples (A1, A2, B1, B2, D1, D2). It can be seen from the figure that with the increase in temperature, the intensity of calcite diffraction peaks gradually decreased. This may be due to the fact that under high temperature conditions, part of the calcite undergoes a relatively intense reaction and is converted into calcium compounds, resulting in a significant weakening of the intensity of its XRD diffraction peaks. Hematite in red mud did not change significantly at different temperatures. The unreacted hematite played a skeleton role in the lightweight aggregates and formed a more robust microstructure together with the gel products, which was consistent with the previous research results [34,35]. In addition, although each sample had the same mineral phases inside, the intensities of their characteristic peaks were significantly different, indicating that different curing methods would not lead to changes in the types of mineral phases, but would affect the quantity of mineral phases [36,37].

#### 3.2.3. FTIR

**Figure 7:**
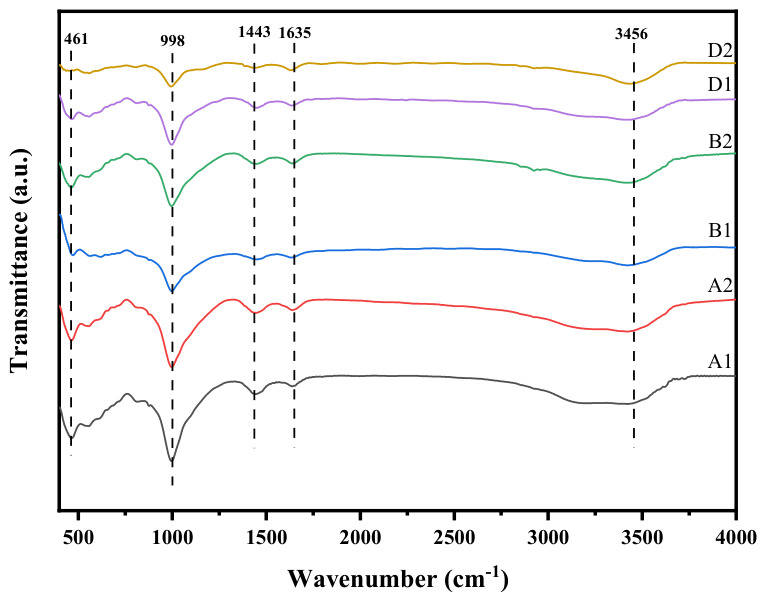
FTIR spectrum of RMLWA.

FTIR analysis was performed on six groups of samples (A1, A2, B1, B2, D1, D2), and the specific results are shown in Figure 7. The absorption peak around 3456 cm^−1^ is attributed to the asymmetric stretching vibration of the O-H bond, indicating the presence of interlayer water in hydroxyl groups [38,39], while the peak at 1635 cm^−1^ corresponds to the bending vibration of the H-O-H bond, indicating the presence of OH groups in gels such as C-S-H [40,41]. The peak at 1437 cm^−1^ corresponds to the symmetric vibration of the C-O bond [33]. In addition, the absorption peaks around 998 and 461 cm^−1^ are considered to be the contraction vibrations of Si(Al)–O groups, which are related to the contents of Si and Al in the structure [42,43]. These results are consistent with the XRD analysis results.

### 3.3. Heavy Metal Leaching Analysis

**Figure 8:**
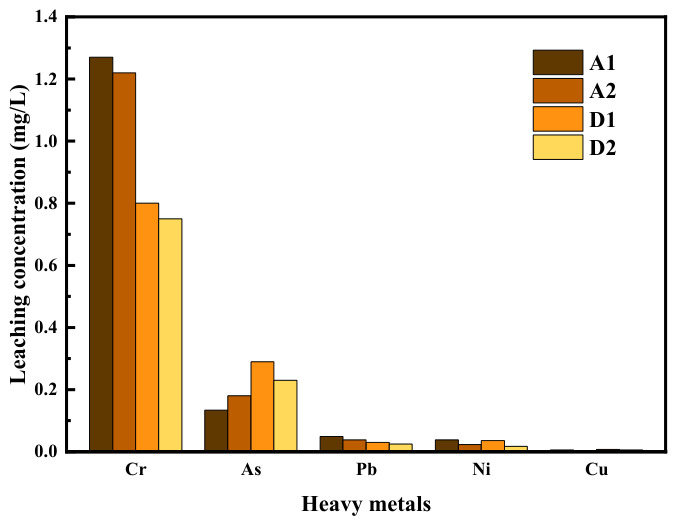
Heavy metal leaching concentration of RMLWA.

Given the presence of heavy metal elements such as Cr, As, Pb, Ni, and Cu in red mud, fly ash, and granulated blast furnace slag, inductively coupled plasma optical emission spectroscopy (ICP-OES) was used in this study to determine the heavy metal leaching concentrations of RMLWA under different curing systems and alkali-activated systems. The environmental safety evaluation was carried out in strict accordance with the limit requirements of the Identification Standards for Hazardous Wastes—Identification of Leaching Toxicity (GB 5085.3-2007) [44], and the results are shown in Figure 8.

Curing systems and types of alkali activators had obvious regulatory effects on the heavy metal leaching behavior. The heavy metal leaching concentrations of steam-cured samples were generally lower than those of standard-cured samples, and the leaching concentrations gradually decreased with the increase in steam curing temperature. Under the same curing conditions, the heavy metal leaching concentrations of samples using sodium silicate solution as activator were lower than those using water as additive. Among them, the curing effect of SS-1.0 combined with high-temperature steam curing was the best. The leaching concentrations of Cr, Pb, Ni, and Cu in sample D2 were the lowest among all samples, which were 0.75 mg/L, 0.025 mg/L, 0.017 mg/L, and 0.006 mg/L, respectively. This is because the largest quantity of gel products such as C-S-H was generated with the densest structure, which solidified the heavy metals. However, the As leaching concentrations of samples D1 and D2 were the highest, which were 0.29 mg/L and 0.23 mg/L, respectively. This is mainly because arsenic in raw materials predominantly exists in stable forms bound to Fe-Mn oxides and encapsulated within glassy phases [45,46]. Heating at 90 °C breaks Fe-As bonds and the network structure of glassy phases, transforming arsenic into soluble arsenate with enhanced mobility [47,48,49]. The high-temperature and high-humidity environment further increases the diffusion coefficient of arsenic ions, driving soluble arsenic to migrate to the aggregate surface via capillary action and accumulate there [47,50,51]. Meanwhile, the highly alkaline environment of the alkali-activated system weakens the adsorption and immobilization of arsenic by iron oxides [52].

### 3.4. Economic Feasibility Analysis

To comprehensively evaluate the economic application potential of RMLWA, the production cost was calculated in this study. Since the material is still in the laboratory stage without large-scale production and field application, the production cost per ton of products with optimal performance was adopted as the evaluation index, so as to provide theoretical support and basic data for its future engineering promotion.

The total production cost of RMLWA mainly consists of raw materials, electricity, and labor costs. Electricity consumption covers granulation, drying, and curing processes, with energy consumption of 5 kWh/t, 24 kWh/t and 96 kWh/t, respectively [53]. Other cost unit prices are derived from previous research literature [54]. According to the unit prices of raw materials listed in Table 4 and the actual consumption of each component during production, the production cost of RMLWA was calculated, and the detailed results are presented in Table 4. The calculated production cost of one ton of RMLWA is 307.03 CNY.

In addition, a comparative analysis with previous studies was performed. Shang et al. [55] fabricated artificial lightweight aggregates using corn straw ash and concrete slurry waste, with a maximum production cost of 666.568 CNY per ton. Liu et al. [12] developed non-fired lightweight aggregates from coal gasification fine slag and blast furnace slag, whose production cost was 380.7 CNY per ton. In comparison, the lightweight aggregate developed in this study presents a lower production cost and possesses distinct price competitiveness.

## 4. Conclusions

In this study, unburned lightweight aggregates were prepared using red mud, fly ash, and granulated blast furnace slag as raw materials, and the effects of curing systems and alkali-activated systems on their properties were explored. The following conclusions were drawn:

(1) Curing temperature and alkali activator had a significant synergistic effect on the physical properties of lightweight aggregates. The effect of steam curing was better than that of standard curing, and the properties improved with the increase in steam temperature. Sodium silicate solution with a modulus of 1.0 was the optimal activator, and sample D2 cured by steam at 90 °C had the best comprehensive performance, with a cylinder compressive strength of 6.92 MPa, a 1 h water absorption rate of 14.8%, a softening coefficient of 0.93, and a void ratio as low as 31.07%, which met the requirements for the use of building lightweight aggregates.
(2) Increasing the curing temperature significantly accelerated the hydration process of the RMLWA system and promoted the abundant formation of cementitious products such as C-S-H gel. These products effectively filled the internal pores and microcracks of aggregates and constructed a dense three-dimensional network skeleton. Such microstructural evolution directly reduced the porosity and water absorption of the material, strengthened the bonding force between particles, and thus improved the mechanical properties and water stability. Meanwhile, the physical encapsulation effect of C-S-H gel provided stable immobilization sites for heavy metal ions. Consequently, the microstructure, physical properties, and environmental safety were synergistically optimized.
(3) For the prepared red-mud-based unburned lightweight aggregates, the leaching concentrations of heavy metals such as Cr, As, Pb, Ni, and Cu all met the limit requirements of the Identification Standards for Hazardous Wastes—Identification of Leaching Toxicity (GB 5085.3-2007).

## 5. Limitations and Future Prospects

This study systematically investigated the preparation technology, microstructural characteristics, physico-mechanical properties, and heavy metal leaching behavior of solid waste-based non-fired lightweight aggregates. However, several limitations were identified in the present work. The long-term service performance of the aggregates was not analyzed in depth, and the structural evolution and performance degradation under harsh service conditions, including freeze-thaw cycles, wet–dry alternations, and acid-base corrosion, were not explored. Furthermore, all experiments were implemented at a small laboratory scale. The practical feasibility of industrial mass production was not evaluated by taking production cost and process parameter adaptability into consideration.

In future studies, emphasis will be placed on the long-term durability and service stability of the material under complex environments. The preparation processes will be optimized and the cost evaluation system will be improved. These efforts will provide more comprehensive data and theoretical basis for the large-scale engineering application of such solid-waste-based lightweight aggregates.

## Figures and Tables

**Table 1:** Chemical compositions of RM, FA, and GGBS.

Composition/wt%	CaO	SiO_2_	Al_2_O_3_	Fe_2_O_3_	SO_3_	MgO	TiO_2_	K_2_O	Na_2_O	LOI^a^
RM	0.45	14.14	16.51	50.12	0.60	0.11	4.83	0.12	4.45	9.17
FA	3.73	48.12	35.15	2.62	1.33	0.38	1.79	2.83	2.15	1.48
GGBS	35.13	31.94	17.57	0.18	2.55	9.25	1.05	0.25	0.80	-

LOI^a^ = loss on ignition.

**Table 2:** Raw Material Mix Proportion and Maintenance Conditions for RMLWA.

Sample	Proportion of Raw Materials (g)			Mixing Water	Curing Condition
	RM	FA	GGBS		
A1	600	300	100	TW	Standard curing at 20 °C for 24 h
A2				SS-1.0	
A3				SS-2.0	
B1	600	300	100	TW	Steam curing at 50 °C for 24 h
B2				SS-1.0	
B3				SS-2.0	
C1	600	300	100	TW	Steam curing at 70 °C for 24 h
C2				SS-1.0	
C3				SS-2.0	
D1	600	300	100	TW	Steam curing at 90 °C for 24 h
D2				SS-1.0	
D3				SS-2.0	

**Table 3:** Test Methods for Physical Properties of Lightweight Aggregates.

Testing Indicators	Mathematical Formulation	Parameter Property
cylinder crush strength	$f_{a}=\frac{p_{1}+p_{2}}{F}$	*f_a_* represents the cylinder compressive strength (MPa), *p*_1_ refers to the applied load (N) at a penetration distance of 20 mm, *p*_2_ indicates the weight of the pressing die (N), and F represents the bearing surface area (F = 10,000 mm^2^).
softening coefficient	$\psi =\frac{f_{1}}{f_{0}}$	*Ψ* denotes the softening coefficient of the artificial lightweight aggregate. *f*_0_ is the crush strength of the dry artificial lightweight aggregate (MPa), while *f*_1_ corresponds to the compressive strength (MPa) in the saturated surface-dry condition after 1 h of immersion.
1-h water absorption rate	$\omega_{a}=\frac{m_{1}-m_{0}}{m_{0}}$	*ω_a_* represents the 1 h-water absorption rate (%) of the artificial aggregate, *m*_0_ denotes the dry weight (g) of the artificial lightweight aggregate, and *m*_1_ represents the weight (g) of the artificial aggregate in the saturated surface-dry state.
apparent density	$\rho_{ap}=\frac{m_{0}\times 1000}{\Delta V}$	*ρ_ap_* is the apparent density (kg/m^3^) of the artificial lightweight aggregate, *m*_0_ is the dry weight (g) of the lightweight aggregate, and ∆*V* is the increment of the liquid level in the measuring cylinder before and after the artificial lightweight aggregate is put in (mL).
bulk density	$\rho_{bu}=\frac{\left(m_{t}-m_{v}\right)\times 1000}{V}$	*ρ_bu_* denotes the bulk density (kg/m^3^) of the artificial lightweight aggregate, *m_t_* is the combined mass (kg) of the aggregate and the volumetric flask, *mᵥ* is the empty flask’s mass (kg), and *V* indicates the flask volume (L).
void ratio	$\nu =\left(1-\frac{\rho_{bu}}{\rho_{ap}}\right)\times 100$	*ν* denotes the void ratio (%), where *ρ_bu_* represents the bulk density (kg/m^3^) of the artificial lightweight aggregate, and *ρ_ap_* represents the apparent density (kg/m^3^) of the artificial lightweight aggregate.

**Table 4:** Production costs of RMLWA.

Category	RMLWA		
	Dosage (kg)	Unit Cost (CNY/t or CNY/kWh)	Cost (CNY)
RM	600	4	2.4
FA	300	90	27
GGBS	100	210	21
Sodium silicate	160	900	144
Water	80	4.81	0.38
Power consumption (kWh)	125	0.818	102.25
Labor	-	10	10
Total			307.03

## Data Availability

The original contributions presented in this study are included in the article. Further inquiries can be directed to the corresponding authors.
